# A study on the correlation between albumin-to-alkaline phosphatase ratio and the severity of pelvic inflammatory disease

**DOI:** 10.3389/fmed.2025.1728185

**Published:** 2026-01-12

**Authors:** Lei Wang, He Yuan, Pengfei Bian, Jinhua Yuan, Yongai Yu

**Affiliations:** Department of Gynecology and Obstetrics, Central Hospital of Dalian University of Technology, Dalian Municipal Central Hospital, Dalian, China

**Keywords:** albumin-to-alkaline phosphatase ratio, biomarker, immune inflammation, pelvic inflammatory disease, prediction

## Abstract

**Objective:**

To evaluate albumin-to-alkaline phosphatase ratio (AAPR) as a pelvic inflammatory disease (PID) severity biomarker and determine PID predictors.

**Methods:**

This retrospective study included ninety-nine patients with PID, as the experimental group, further classified by severity into mild and severe PID groups. The control group included forty-nine patients who underwent elective surgery for non-inflammatory tubal-ovarian masses in the same period. Relationships between PID severity and general information, vital signs, laboratory indices, and imaging characteristics among the three groups were analyzed. Independent PID severity predictors were determined using multivariate regression analysis.

**Results:**

(1) Significant differences were observed in body temperature, heart rate, respiratory rate, white blood cell count, neutrophil percentage, absolute neutrophil count, alkaline phosphatase, fibrinogen, neutrophil-to-lymphocyte ratio, and platelet-to-lymphocyte ratio among the mild PID, severe PID, and control groups (*P* < 0.001). These indices were positively correlated with disease severity. Meanwhile, absolute lymphocyte count, serum albumin level, and AAPR significantly decreased with disease progression (*P* < 0.001). (2) Severe PID group exhibited elevated C-reactive protein, procalcitonin, and pelvic mass diameter compared to the mild group (*P* < 0.05). (3) Multivariate analysis confirmed AAPR as an independent predictor of PID severity (OR = 0.0041, 95% CI: 0.0003–0.0519, *P* < 0.001). (4) ROC curve analysis suggested that AAPR may serve as an indicator for diagnosing severe PID, with an AUC of 0.808 (95% CI: 0.733–0.882). The AUC of AAPR combined with neutrophil percentage was 0.947 (95% CI: 0.914–0.980). (5) The study highlights AAPR's high potential as a biomarker for dynamic treatment monitoring.

**Conclusions:**

AAPR was negatively correlated with PID severity and was identified as an independent PID severity predictor, providing a new reference for clinical disease monitoring.

## 1 Introduction

Pelvic inflammatory disease (PID) encompasses upper genital tract infections, such as endometritis, salpingitis, tubo-ovarian abscess, and pelvic peritonitis, among which salpingitis and tubo-ovarian abscess predominate (1). The main pathogens involved in these infections are sexually transmitted microorganisms such as *Neisseria gonorrhoeae* and *Chlamydia trachomatis* (2). PID predominantly affects sexually active women of reproductive age, while it is rare in premenarchal, sexually inactive, and postmenopausal women (1). Untreated PID can lead to infertility, ectopic pregnancy, or chronic pelvic pain (2, 3). In severe cases, it may progress to systemic inflammatory response syndrome (SIRS) or even sepsis, posing a significant threat to female reproductive health (1, 4).

Albumin (ALB) reflects the body's inflammatory and nutritional status, and performs multiple physiological roles including in the regulation of osmotic pressure, transport and metabolism, and antioxidant activity (5–7). During inflammation, ALB levels decrease through multiple mechanisms. Inflammatory mediators such as interleukin-1, interleukin-6, and tumor necrosis factor suppress hepatic ALB synthesis, reducing its levels in serum (8). Inflammatory states increase capillary endothelial permeability, causing albumin to leak from blood vessels into tissue spaces, decreasing intravascular albumin content, and inducing tissue edema. The body's hypermetabolic state during inflammation accelerates albumin catabolism, further reducing the levels of ALB in the blood (9). Clinically, albumin levels are used to assess the severity and prognosis of inflammatory diseases. Hypoalbuminemia is indicative of severe inflammation, guiding prognostic assessment (10). Alkaline phosphatase (ALP) is ubiquitous in liver, bone, and intestinal tissues. Inflammatory stimuli induce its synthesis and secretion in multiple cell types (11). Inflammation alters the permeability of the cell membrane, facilitating the release of ALP from cells into the bloodstream and thereby increasing serum ALP levels (12). Microenvironmental changes during inflammation may also enhance the activity of serum ALP (10). Clinical detection of serum ALP levels can facilitate the diagnosis, monitoring, and prognostic evaluation of inflammatory diseases.

Recent studies have established the Albumin-to-Alkaline Phosphatase Ratio (AAPR) as a composite biomarker that reflects both nutritional status and systemic inflammation (11, 13, 14). It has demonstrated significant prognostic value for survival in various malignancies and cardiovascular diseases, including hepatocellular carcinoma and cholangiocarcinoma (11, 15–19). Nevertheless, its role in non-malignant inflammatory disorders, particularly pelvic inflammatory disease (PID), has not been investigated. This study posits that AAPR could serve as a novel, practical biomarker for assessing PID severity, based on the correspondence between PID's pathophysiology and the physiological processes captured by AAPR. PID is characterized by an upper genital tract inflammatory response initiated by pathogenic infection. In severe or prolonged cases, the release of pro-inflammatory cytokines can lead to systemic inflammation, resulting in decreased hepatic albumin synthesis, reduced protein intake, and increased protein loss. Concurrently, inflammatory stress may drive a compensatory rise in alkaline phosphatase. As AAPR inherently represents the balance between inflammatory stress and nutritional-metabolic status, we hypothesize it may be a useful indicator of PID severity. The aim of this study is to evaluate the independent association and diagnostic performance of AAPR in PID, thereby addressing a current gap in clinical assessment tools.

## 2 Materials and methods

### 2.1 Subjects

A retrospective analysis was conducted on 99 patients with PID, as an experimental group. These patients were stratified by SIRS manifestations into a mild group (*n* = 53) and a severe group (*n* = 46). In addition, 49 patients with non-inflammatory tubo-ovarian masses who underwent elective surgery at the Department of Gynecology of the same hospital during the same period were selected as a control group. Ethical approval for this study was obtained from the institutional ethics committee and written informed consent was waived.

### 2.2 Inclusion and exclusion criteria

The inclusion criteria for this study were as follows: (1) diagnosed with PID in accordance with the 2021 diagnostic criteria set by the Center for Disease Control and Prevention (CDC, USA) and (2) availability of complete clinical records. Meanwhile, the exclusion criteria for the study group and the control group are as follows: (1) complicated with severe dysfunction of vital organs (heart, lungs, liver, kidneys) not caused by inflammation: Cardiac insufficiency: NYHA Class III or higher. Hepatic insufficiency: Child-Pugh Class B or C; or ALT/AST > 3 × ULN. Renal insufficiency: eGFR < 60 mL/min/1.73 m^2^. Respiratory insufficiency: Requires long-term home oxygen therapy, or PaO < 60 mmHg (on room air); (2) history of malignant tumor; (3) Continuous use of corticosteroids (equivalent to prednisone > 10 mg daily), immunosuppressants, or biologics within the past 3 months; (4) pregnant or lactating women; (5) poor treatment compliance or withdrawal from treatment midway through it; (6) incomplete clinical data; (7) exclude patients with other conditions that may significantly affect ALB/ALP levels, such as acute drug/alcohol-induced liver injury, acute viral hepatitis, biliary obstruction, severe thyroid dysfunction, or severe malnutrition (albumin < 25 g/L unrelated to inflammation).

### 2.3 Methods

The electronic medical records were used to gather the following information: (1) basic information: age, body mass index (BMI), parity and gravidity, and comorbidities (diabetes, hypertension, cardiovascular, and neurological disorders, etc.); (2) Laboratory indicators, including white blood cell count (WBC), neutrophil percentage (NEUT%), absolute neutrophil count (ANC), absolute lymphocyte count (ALC), platelet count (PLT), C-reactive protein (CRP), procalcitonin (PCT), ALB, ALP, and fibrinogen (FIB), were measured using venous blood samples obtained within 24 h of admission and prior to the administration of any treatment. Liver function was serially monitored throughout the current treatment course via blood sampling. Serum ALB and ALP levels were measured using a fully automated biochemical analyzer (Model: ADVIA Chemistry XPT, Manufacturer: Siemens Healthcare Diagnostics Inc.). ALB was determined by the bromocresol green method, and ALP by the disodium phenylphosphate method. The laboratory strictly implements quality control, performing daily internal quality control with inter-assay CVs < 5%, and regularly participates in external quality assessments; and (3) imaging parameters, including pelvic mass size, unilateral/bilateral involvement, and depth of pelvic effusion.

The diagnosis of PID was based on the 2021 diagnostic criteria recommended by the Centers for Disease Control and Prevention (CDC, USA) (2) as follows: (1) minimum indicators: cervical motion/uterine/adnexal tenderness; (2) supportive indicators: fever (>38.8 °C oral temperature), cervical purulence or bleeding susceptibility, vaginal WBCs on saline microscopy, elevated inflammatory markers (ESR/CRP), laboratory-confirmed cervical *N. gonorrhoeae* or *C. trachomatis* infection; and (3) confirmatory evidence: endometrial biopsy confirming endometritis, transvaginal ultrasound or MRI showing tubal thickening, hydrosalpinx, with or without pelvic effusion or tubo-ovarian mass, and laparoscopic findings consistent with PID.

Diagnostic criteria for SIRS (20) were as follows: (1) fever or hypothermia (body temperature >38 °C or < 36 °C); (2) heart rate > 90 bpm; (3) respiratory rate >20 bpm or PaCO_2_ < 32 mmHg; and (4) WBC > 12.0 × 10^9^/L or < 4.0 × 10^9^/L, or immature granulocytes > 10%. Diagnosis was established when two or more of the above criteria were met. Severe PID was defined exclusively based on SIRS criteria.

AAPR: The ratio of serum albumin-to-alkaline phosphatase is known as the AAPR.
Neutrophil-to-lymphocyte ratio (NLR): The ratio of the ANC to the ALC in peripheral blood.
Platelet-to-lymphocyte ratio (PLR): The ratio of the platelet count to the ALC in peripheral blood.

### 2.4 Statistical analysis

Statistical analysis was performed using SPSS 27.0 and GraphPad Prism 9.5. Normally distributed continuous variables are presented as mean ± standard deviation, while non-normally distributed data as median (IQR). For multi-group comparisons of continuous variables, ANOVA or Kruskal–Wallis tests were applied. Categorical data are expressed as number of cases (*n*) and percentage (%), compared using the Chi-square (χ^2^) test or Fisher's exact test, with *post-hoc* pairwise comparisons conducted when significant differences were detected. Univariate and multivariate logistic regression were employed to identify independent predictors of pelvic inflammatory disease, with the regression coefficient (B), odds ratio (OR), and corresponding 95% confidence interval (95% CI) used to assess the relationship and impact of each factor on disease probability. A two-tailed P-value < 0.05 was considered statistically significant. A binary logistic regression model was established to create a combined diagnostic indicator using AAPR and neutrophil percentage (NEUT%). Disease severity served as the dependent variable, while 1/AAPR and NEUT% were independent variables. The model's core formulas are: Linear Predictor (LP): LP = β_0_+ (β_1_× (1/AAPR)) + (β_2_ × NEUT%); Predicted Probability (*P*): *P* = e^*LP*^/(1 + e^*LP*^). The predicted probability (*P*) was saved as a new continuous variable (the combined diagnostic indicator) and subsequently subjected to Receiver Operating Characteristic (ROC) curve analysis to evaluate its comprehensive diagnostic performance for severe pelvic inflammatory disease. For dynamic AAPR monitoring data, paired *t*-test or Wilcoxon signed-rank test were used.

## 3 Results

### 3.1 Comparison of patients' clinical/demographic characteristics

A total of 148 subjects were enrolled in this study, including 99 patients with PID in the experimental group, who were divided into mild (*n* = 53) and severe groups (*n* = 46) according to the disease severity, and 49 patients with non-inflammatory tubo-ovarian masses in the control group. Analysis of the demographic characteristics showed no significant differences in age, BMI, gravidity/parity, comorbidities (hypertension, diabetes, cardiovascular diseases, etc.), depth of pelvic effusion, or unilateral/bilateral adnexal mass location among the three groups (*P* > 0.05). Moreover, comparison of the clinical characteristics showed statistically significant differences in body temperature, heart rate, and respiratory rate among the three groups (*P* < 0.001). Moreover, compared with the severe group, both the control and mild groups had significantly lower body temperature and heart rate (*P* < 0.001). No significant differences in these parameters were found between the control and mild groups themselves (*P* > 0.05). A significantly lower respiratory rate was found in the control group relative to those in the mild and severe groups (*P* < 0.05). In contrast, the respiratory rates of the mild and severe groups did not differ significantly (*P* > 0.05). Compared with the severe and mild groups, inflammatory masses were significantly larger in the severe group (*P* < 0.05), as shown in Table 1.

**Table 1:** Demographic and clinical characteristics of patients.

**Characteristics**	**Control group (*n =* 49)**	**Mild group (*n =* 53)**	**Severe group (*n =* 46)**	***F*/χ^2^/*H*/*Z***	***P***	**Control vs. mild group**		**Mild vs. severe group**	
						***Z***	***P***	***Z***	***P***
Age (years)	39.33 ± 13.20	42.28 ± 12.52	44.91 ± 12.42	2.295	0.104				
BMI (kg/m^2^)	22.19 ± 4.64	23.48 ± 4.35	24.08 ± 3.43	2.529	0.083				
Gravidity (times)	1(0.5, 2.0)	2(1, 3)	2(1, 2.25)	3.538	0.17				
Parity (times)	1(0, 1)	1(0, 1)	1(1, 1)	2.521	0.284				
Temperature (°C)	36.3(36.3, 36.5)	36.5(36.2, 36.9)	37.7(36.8, 38.4)	44.227	< 0.001^*^	−1.738	>0.05	−6.656	< 0.001^*^
Heart rate (bpm)	80(76, 88)	80(78, 97)	100(93.5, 110)	42.754	< 0.001^*^	−0.895	>0.05	−5.957	< 0.001^*^
Respiratory rate (bpm)	18(18, 20)	20(18, 20)	20(20, 20)	25.667	< 0.001^*^	−3.455	0.002^*^	−1.756	0.239
Effusion depth (mm)	4.86 ± 12.16	16.32 ± 19.05	7.98 ± 17.07	0.75	0.785				
Complication				0.061	0.805				
None	24(49.0%)	34(64.2%)	15(32.6%)						
Yes	25(51.0%)	19(35.8%)	31(67.4%)						
Mass size (cm)		5.4(2.85, 6.0)	6.4(4.6, 8.1)	−2.405	0.016^*^				
Unilateral or bilateral				0.536	0.765				
Unilateral		27(50.9%)	21(45.7%)						
Bilateral		17(32.1%)	18(39.1%)						
None		9(17%)	7(15.2%)						

^*^Statistically significant difference. *Post-hoc* pairwise comparisons were performed with Bonferroni correction, with *P* values adjusted accordingly.

### 3.2 Comparison of laboratory indices among patient groups

Bonferroni-adjusted *post hoc* comparisons revealed that most indicators remained statistically significant between groups. Specifically, with increasing disease severity, white blood cell parameters (WBC, NEUT%, ANC), coagulation markers (FIB), inflammatory ratios (NLR, PLR) and ALP showed a stepwise increasing trend, while ALC and AAPR displayed a stepwise decreasing trend—all with statistically significant differences (adjusted *P* < 0.001 or *P* < 0.05). Furthermore, within the patient groups, severe cases exhibited significantly higher levels of CRP and PCT compared to mild cases (*P* < 0.001). In contrast, although PLT and ALB showed numerical increases and decreases, respectively, their intergroup differences did not reach statistical significance after Bonferroni correction as shown in Table 2.

**Table 2:** Comparison of laboratory indicators among patients.

**Indicators**	**Control group (*n =* 49)**	**Mild group (*n =* 53)**	**Severe group (*n =* 46)**	***F*/*H*/*Z***	***P***	**Control vs. mild group**		**Mild vs. severe group**	
						**Z/t**	**P**	**Z/t**	**P**
WBC (×10^9^/L)	5.66(5.05, 6.23)	8.19(6.76, 11.29)	15.52(12.67, 18.24)	83.977	< 0.001^*^	−5.754	< 0.001^*^	−6.02	< 0.001^*^
NEUT (%)	54.22 ± 8.78	72.13 ± 11.63	85.96 ± 6.23	140.159	< 0.001^*^	−8.820	< 0.001^*^	−7.504	< 0.001^*^
ANC(×10^9^/L)	3.02(2.54,3.68)	5.71(4.5,8.86)	13.16(10.25,15.81)	93.06	< 0.001^*^	−6.507	< 0.001^*^	−5.918	< 0.001^*^
ALC(×10^9^/L)	2.02(1.75, 2.47)	1.51(1.21, 2.02)	1.17(0.75, 1.73)	34.444	< 0.001^*^	−3.671	0.002^*^	−3.014	0.023^*^
PLT (×10^9^/L)	239(194, 292)	273(203, 361.5)	312(228, 360.25)	7.757	0.021^*^	−1.655	0.302	−1.221	0.682
ALB (g/L)	42.41 ± 2.95	40.50 ± 3.77	38.74 ± 4.83	10.53	< 0.001^*^	2.837	0.044^*^	2.033	0.08
ALP (U/L)	56(46, 62)	71(58.5, 87)	82.5(69.75, 121.5)	53.221	< 0.001^*^	−4.717	< 0.001^*^	−3.376	0.007^*^
AAPR(g/U)	0.74(0.67, 0.95)	0.56(0.47, 0.69)	0.43(0.28, 0.58)	59.062	< 0.001^*^	−5.327	< 0.001^*^	−3.516	0.008^*^
FIB (g/L)	2.76(2.50, 2.97)	4.65(3.30, 6.16)	6.89(5.73, 8.56)	89.484	< 0.001^*^	−6.795	< 0.001^*^	−4.901	< 0.001^*^
NLR	1.51(1.19, 1.91)	4.08(2.22, 6.39)	10.93(6.46, 16.22)	95.925	< 0.001^*^	−6.554	< 0.001^*^	−6.023	< 0.001^*^
PLR	114(89, 156)	164(125, 261)	246(178, 402)	46.766	< 0.001^*^	−3.975	< 0.001^*^	−3.634	< 0.001^*^
CRP (mg/L)		15.15(3.985, 95.835)	139.845(95.07, 254.66)	−5.472	< 0.001^*^				
PCT (ng/ml)		0.02(0.01, 0.135)	0.52(0.11, 2.685)	−4.397	< 0.001^*^				

^*^Statistically significant difference. *Post-hoc* pairwise comparisons were performed with Bonferroni correction, with P values adjusted accordingly.

### 3.3 Analysis of factors influencing PID severity

Univariate and multivariate logistic regression analyses assessed factors potentially influencing PID severity. After conducting univariate analysis on all variables, fibrinogen was removed. The remaining variables then underwent collinearity diagnostics and stepwise backward regression, after which key predictive factors were included in the multivariate analysis. Multivariate analysis revealed the following two independent predictors of PID severity: neutrophil percentage (adjusted OR = 1.229, 95% CI: 1.156–1.306, *P* < 0.001) and AAPR (adjusted OR = 0.0041, 95% CI: 0.0003–0.0519, *P* < 0.001), as shown in Table 3.

**Table 3:** Logistic regression results for PID severity factors.

**Factors**	**Univariate analysis**		**Multivariate analysis**	
	**OR (95% CI)**	***P***	**OR (95% CI)**	***P***
NEUT (%)	1.201(1.150–1.254)	< 0.001^*^	1.229(1.156–1.306)	< 0.001^*^
ALC (×10^9^)	0.255(0.153–0.425)	< 0.001^*^	2.104(0.964–4.594)	0.062
AAPR(g/U)	0.00088(0.00012–0.00634)	< 0.001^*^	0.0041(0.0003–0.0519)	< 0.001^*^

^*^Statistically significant difference.

### 3.4 Evaluation of AAPR's ability to predict severe PID

**Figure 1:**
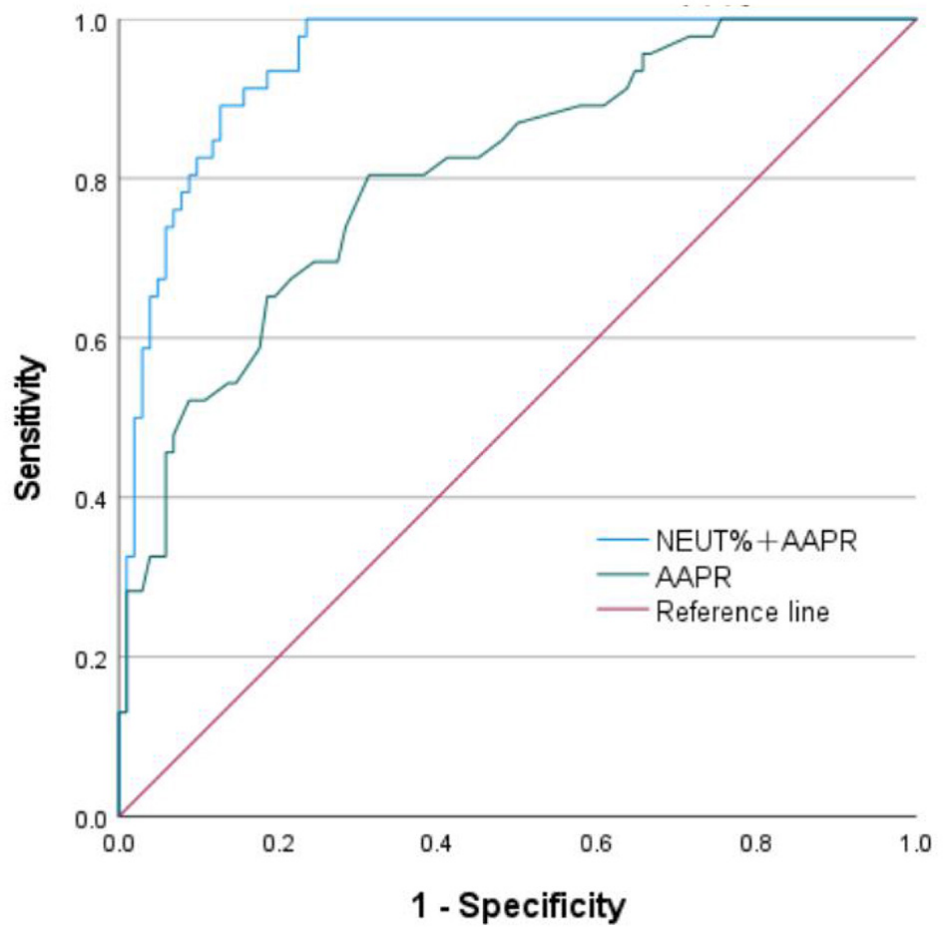
Receiver operating characteristic (ROC) curve comparing “NEUT% + AAPR” and “AAPR” with a reference line. The curve demonstrates sensitivity versus 1-specificity. The blue line for “NEUT% + AAPR” shows better diagnostic performance with a steeper ascent and higher peak than the green “AAPR” line. The reference line in red represents no diagnostic ability.

Using logistic regression, a weighted combination of 1/AAPR and NEUT% generates a predictive probability as a unified diagnostic indicator for receiver operating characteristic ROC analysis. Using severe PID as the outcome, ROC curve was used to analyze the diagnostic value of AAPR. The results showed that the area under the ROC curve (AUC) of AAPR for diagnosing severe PID was 0.808 (95%CI: 0.733–0.882). The optimal cut-off value of AAPR determined by the maximum Youden's index was 0.595. With this cut-off value, the sensitivity and specificity of AAPR for diagnosing severe PID were 80.4% and 68.6%, respectively. The AUC of AAPR combined with neutrophil percentage was 0.947 (95%CI: 0.914–0.980), with a sensitivity of 100% and a specificity of 76.5%, as shown in Table 4 and Figure 1.

**Table 4:** Clinical value of single indicator and combined two indicators in predicting severe PID.

**Indicators**	**AUC**	**95%CI**	**Sensitivity**	**Specificity**
AAPR	0.808	0.733–0.882	0.804	0.686
NEUT%	0.924	0.884–0.965	0.978	0.775
AAPR + NEUT%	0.947	0.914–0.980	1	0.765

### 3.5 AAPR dynamic changes

**Figure 2:**
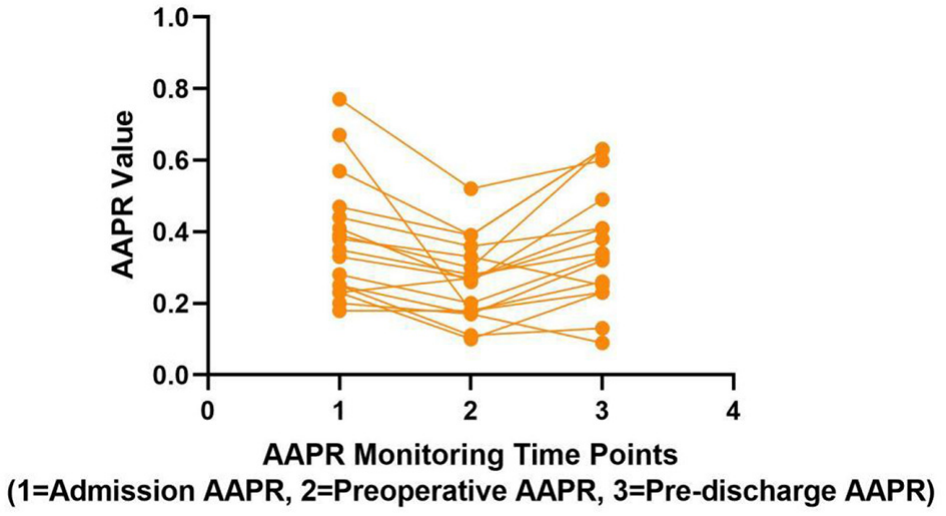
Longitudinal AAPR monitoring spaghetti plot. Line graph showing AAPR values across three monitoring time points: Admission, Preoperative, and Pre-discharge. Each point is connected by lines, with values ranging mostly between 0.2 and 0.8.

We collected 17 cases of pelvic inflammatory disease patients who failed anti-inflammatory therapy upon admission, subsequently underwent surgery, and had their AAPR monitored upon admission, before surgery, and prior to discharge. Statistical analysis revealed that during the pre-operative phase, the median AAPR at admission 0.35(0.24, 0.46) was significantly higher than the pre-operative median AAPR 0.27(0.18,0.35) (paired Wilcoxon test, *P* < 0.001). In the post-operative phase, the pre-operative mean AAPR level (0.26 ± 0.11) was significantly lower than the mean AAPR at discharge (0.37 ± 0.17) (paired *t*-test, *P* < 0.001), as shown in Tables 5, 6. Our exploratory analysis preliminarily suggests that AAPR may exhibit a unique “V-shaped” dynamic trajectory during the perioperative period, which corresponds with improvements in the patient's clinical course. This trend is visually represented in Figure 2. These preliminary findings provide conceptual support for the potential utility of AAPR as a dynamic monitoring tool for treatment response.

**Table 5:** Paired comparisons of dynamic AAPR changes during the deterioration phase.

**Factors**	**Admission AAPR**	**Preoperative AAPR**	***Z***	***P***
AAPR	0.35(0.24, 0.46)	0.27(0.18, 0.35)	−3.418	< 0.001

**Table 6:** Paired comparisons of dynamic AAPR changes during the recovery phase.

**Factors**	**Preoperative AAPR**	**Discharge AAPR**	***t***	***P***
AAPR	0.26 ± 0.11	0.37 ± 0.17	−4.166	< 0.001

## 4 Discussion

PID refers to an infection in the female upper reproductive tract, which affects the endometrium, fallopian tubes, ovaries, or pelvic peritoneum. Its pathogenesis primarily involves infection by microorganisms ascending through the genital tract. Epidemiological analyses have revealed that it shows a high incidence among sexually active women. Common risk factors include an active sex life, sexual partners with sexually transmitted diseases, lower-genital-tract infections, infections after intrauterine surgical procedures, and poor menstrual hygiene (1, 2, 4). Clinical manifestations are diverse, with the main symptoms including persistent lower abdominal pain and increased vaginal discharge. Associated symptoms may involve multiple systems, such as menstrual disorders (irregular cycles, abnormal menstrual flow), gastrointestinal irritation (nausea, vomiting, diarrhea, tenesmus), and urinary symptoms (frequency, urgency, dysuria) (2, 3). Gynecological examination may reveal purulent foul-smelling vaginal discharge; cervical congestion, edema, or purulent discharge; cervical motion tenderness; uterine body or adnexal tenderness; and pelvic mass formation in cases of abscess (2, 3, 21). Inflammatory biomarkers such as peripheral blood WBC, neutrophil percentage, CRP, and PCT are key indicators for assessing the severity and progression of inflammatory disease (22–24). This study's intergroup comparative analysis showed significantly higher levels of WBC, neutrophil percentage, CRP, and PCT in severe PID patients compared with that in mild cases, with values in the mild group also being significantly higher than in the control group. These results strongly align with those in similar previous studies.

PID can be classified into mild and severe forms, and can even progress to sepsis. At present, there are no specific indicators in clinical practice to predict disease progression. The methods for assessing the severity of infection clinically, such as the Oxford Acute Disease Severity Score and Sequential Organ Failure Assessment, involve a large number of indicators with low specificity and sensitivity (10). This study explored the indicators affecting the severity of PID, with the results showing that neutrophil percentage, CRP, FIB, NLR, and PLR levels were positively correlated with the severity of PID, aligning with previous studies (22). In addition, multivariate analysis showed that neutrophils and AAPR were independent predictors of PID severity.

AAPR as a novel biomarker possesses the dual functions of reflecting nutritional status and inflammatory level (25). Studies performed to date have confirmed its clinical value in assessing the prognosis of various diseases and malignant tumors (26–30). However, in the field of PID, although AAPR shows potential as an inflammatory marker, to the best of our knowledge there has been no research on its correlation with PID progression, highlighting the urgent need for in-depth investigation to clarify its potential for clinical application. Through the integration of clinical data and advanced statistical analysis, this study identified AAPR as an independent predictor of PID severity, demonstrating its clinical value in predicting disease progression. The findings mirror previous research on inflammatory diseases. For example, Feng Zhang confirmed in his study on patients with hepatocellular carcinoma and cholangiocarcinoma that AAPR is an independent prognostic indicator for these two cancers (15). Aya Katasako and others confirmed in a hemodialysis cohort that elevated ALP levels were positively correlated with bacteremia risk, supporting ALP's clinical value as a marker of infection-related inflammation (31). Liu's team also revealed an independent correlation between APAR (alkaline phosphatase-to-albumin ratio) and sepsis prognosis in a retrospective study. Elevated APAR was significantly associated with higher mortality, demonstrating independent predictive ability in multivariate Cox regression models (10). Previous studies suggested that changes in AAPR essentially reflect the imbalance between inflammatory response and nutritional metabolism in the body. Tumor burden induces chronic inflammation and consumes albumin, leading to decreased AAPR. As an acute infectious disease, PID should theoretically cause abnormal changes in AAPR, which provides a mechanistic basis for exploring the association between AAPR and PID.

This study confirms that AAPR is significantly associated with the severity of PID, supporting our initial hypothesis. The underlying pathophysiological rationale is multifaceted. Severe or persistent PID can induce a systemic inflammatory response via the release of pro-inflammatory cytokines such as IL-6 and TNF-α, triggered by pathogen-associated molecular patterns activating TLR and downstream NF-κB/MAPK pathways (17, 32). Within this state, IL-6 suppresses hepatic albumin synthesis while promoting acute-phase protein production, and increased vascular permeability exacerbates hypoalbuminemia (10). Concurrently, symptoms like chronic pelvic pain and anorexia contribute to malnutrition, further lowering albumin levels. The elevation in ALP may stem from release by neutrophils infiltrating the pelvic cavity during infection, or from the inflammatory injury and repair processes of the affected fallopian tube and ovarian tissues (12, 32, 33). Albumin is a recognized biomarker of critical illness, and hypoalbuminemia indicates poor prognosis in systemic inflammation, while ALP levels correlate with conventional inflammatory markers (34, 35). Therefore, a decreased AAPR in severe PID effectively integrates these dual phenomena: inflammation-driven albumin suppression and ALP elevation. Unlike prior research focused on oncology, our findings highlight AAPR's utility in a acute inflammatory gynecological condition, offering a simple, integrative index for severity stratification in clinical practice.

## 5 Limitations

Several limitations of this study should be acknowledged. First, the retrospective design makes it difficult to fully eliminate selection bias and confounding factors. Second, in this study, the diagnosis of severe PID relied solely on SIRS criteria, which has inherent limitations; future studies could incorporate additional clinical indicators for more precise stratification. Due to the relatively limited sample size, the high diagnostic performance demonstrated by the combination of AAPR and neutrophil percentage may be subject to some degree of optimistic bias within this cohort. Although we employed multiple methods for variable selection, the multivariable analysis may still carry a risk of overfitting. Our preliminary findings provide conceptual support for the potential value of AAPR as a dynamic monitoring tool for treatment response. These limitations highlight the need for validation in larger-scale, prospectively designed studies to confirm reliability and clinical utility, and to more accurately assess the potential of AAPR as an inflammatory biomarker.

## 6 Conclusion

This study confirms that the AAPR is an independent predictor of PID severity. It demonstrates an independent negative correlation with PID severity, where a lower AAPR indicates more pronounced systemic inflammatory responses and poorer clinical outcomes. Our preliminary investigation suggests that the AAPR has good diagnostic value for severe PID, and its dynamic changes hold predictive and prognostic significance in assessing disease progression. The AAPR can serve as a standalone inflammatory biomarker or be combined with other infection-related indicators to assist clinicians in early risk stratification and severity assessment for PID patients, thereby optimizing clinical decision-making.

## Data Availability

The original contributions presented in the study are included in the article/supplementary material, further inquiries can be directed to the corresponding author.
